# Supplementary material for: Structural basis of Nrd1–Nab3 heterodimerization
Source: Life Sci Alliance. 2022 Jan 12;5(4):e202101252. doi: 10.26508/lsa.202101252 (PMC8761494; doi:10.26508/lsa.202101252)
Supplement: Supplementary file 1 [file LSA-2021-01252_TableS1.docx]

Table S1. Summary of NMR restraints and structural calculation statistics for Nab3_191-261_ (PDB:7PRE) and Nrd1_158-221_-Nab3_203-261_ (PDB:7PRD) solution structures.

| NMR experimental restraints | Nab3_191-261_ | Nrd1_158-221_-Nab3_203-261_ |
| --- | --- | --- |
| *NOE-derived* |  |  |
| Intraresidue | 157 | 328 |
| Sequential | 58 | 386 |
| Medium-range (1 < i-j < 4) | 112 | 635 |
| Long-range (i-j > 4) | 22 | 562 |
| Total per residue | 5 | 15 |
| Pseudo intermolecular (Nrd1 to Nab3 in chimera) | - | 305 |
| *TALOS+ obtained restraints* |  |  |
| φ angle restraints | - | 85 |
| ψ angle restraints | - | 92 |
| **Structure statistics** |  |  |
| *Violations* |  |  |
| Distance* | 3±1 | 3 ± 1 |
| Maximum distance violation (Å) | 0.31 | 0.58 |
| Angle** | - | 2 ± 1 |
| Maximum angle violation (º) | - | 11.9 |
| *Ramachandran Plot analysis (range)* | *(208-219)* | *(167-274)* |
| Residues in most favoured regions | 98 ± 4 % | 92 ± 2 % |
| Residues in additionally allowed regions | 0 ± 0 % | 6 ± 2 % |
| Residues in disallowed regions | 2 ± 4 % | 2 ± 1 % |
| *Averaged RMSD to mean structure (range)* |  | *(Nrd1:169-220-Nab3:201-252)* |
| N, CO, Cα (Å) (± SD) | *** | 0.81 ± 0.14 |
| All heavy (Å) (± SD) | *** | 0.97 ± 0.11 |

*Averaged value per structure of distance violations > 0.20 Å ± SD.

** Averaged value per structure of total angle violations

*** No calculation was performed because the structure has not a well-defined ternary fold
